# Establishment of a retinal hypoxia organ culture model

**DOI:** 10.1242/bio.025429

**Published:** 2017-07-15

**Authors:** S. Schnichels, M. Blak, J. Hurst, T. Dorfi, K. U. Bartz-Schmidt, F. Ziemssen, M. S. Spitzer, M. Schultheiss

**Affiliations:** 1 Centre of Ophthalmology, University Eye Hospital Tübingen, Elfriede-Aulhorn-Str. 7, D-72076 Tübingen, Germany; 2 Department of Ophthalmology, Katharinen-Hospital Klinikum Stuttgart, Kriegsbergstr. 60, 70174 Stuttgart, Germany; 3 Department of Ophthalmology, University Medical Center Hamburg-Eppendorf (UKE), Martinistraβe 52, Hamburg, Germany

**Keywords:** Hypoxia, Retina, RGCs, Organ culture, Glutamate excitotoxicity

## Abstract

Hypoxia plays an important role in several retinal diseases, especially in central retinal artery occlusion (CRAO). Although CRAO has been known for over a hundred years, no cure or sufficient treatment is available. Potential therapies are being evaluated in several *in vivo* models or primary cultures. However, *in vivo* models or primary cultures are very time-consuming, expensive, and furthermore several therapies or agents cannot be tested. Therefore, we aimed to develop a standardized organotypic *ex vivo* retinal hypoxia model. A chamber was developed in which rat retinal explants were incubated for different hypoxia durations. Afterwards, the retinas were adjusted to normal air and incubated for 24, 48 or 72 h under standard conditions. To analyze the retinal explants, and in particular the retinal ganglion cells (RGC) immunohistology, western blot and optical coherence tomography (OCT) measurements were performed. To compare our model to a standardized degeneration model, additional retinal explants were treated with 0.5 and 1 mM glutamate. Depending on hypoxia duration and incubation time, the amount of RGCs decreased and accordingly, the amount of TUNEL-positive RGCs increased. Furthermore, β-III-tubulin expression and retinal thickness significantly decreased with longer-lasting hypoxia. The reduction of RGCs induced by 75 min of hypoxia was comparable to the one of 1 mM glutamate treatment after 24 h (20.27% versus 19.69%) and 48 h (13.41% versus 14.41%) of incubation. We successfully established a cheap, standardized, easy-to-use organotypic culture model for retinal hypoxia. We selected 75 min of hypoxia for further studies, as approximately 50% of the RGC died compared to the control group after 48 h.

## Abstract (precis)

**Summary:** An easy-to-use *ex vivo* retinal hypoxia model is introduced that reliably induced retinal damage on a morphological (retinal thickness), and molecular (protein expression and apoptotic markers) level.

## INTRODUCTION

Hypoxia plays an important role in several ophthalmologic diseases such as central retinal artery occlusion (CRAO) (Hayreh et al., 2004), anterior ischemic optic neuropathy (AION) (Hayreh, 2009a,b), glaucoma (Schmidl et al., 2011; Cherecheanu et al., 2013), retinal vein occlusion (Terui et al., 2011; Hayreh, 2005), and diabetic retinopathy (Wessel et al., 2012). In the mentioned diseases retinal hypoxia/ischemia results in irreversible visual impairment and blindness (Osborne et al., 2004). Retinal ganglion cells (RGCs) are especially sensitive to hypoxic stress (Kergoat et al., 2006; Schmid et al., 2014); and therefore, RGCs are crucial in the pathophysiology of these diseases and are of great interest in research.

Several models are used to investigate retinal hypoxia. Since the RGC-5 cell line dedifferentiated (Van Bergen et al., 2009; Schnichels et al., 2012b; Schultheiss et al., 2012; Wood et al., 2010; Krishnamoorthy et al., 2013) only the use of primary RGCs remains for cell culture experiments. However, the preparation of primary RGCs is both very time- and animal-consuming, the complexity of a whole organ system is lacking and the RGCs are extracted out of their natural tissue network. Therefore, the RGCs lose all of their natural cell-to-cell contacts, most of their dendrites, and suffer from axotomy. The cells in a RGC primary cell culture are therefore hardly comparable with the cells in a living tissue.

Explants of human, porcine and rodent retinal tissue have been established and used to study retinal ganglion cell survival and other disease mechanisms (Niyadurupola et al., 2011; Bull et al., 2011; Pattamatta et al., 2016; Xin et al., 2007; Kuehn et al., 2016, 2017). The benefit of organ cultures in comparison to cell culture is that the complex structure of the retina, with all the connections and different cell types, is maintained and therefore it better reflects the physiological situation (Januschowski et al., 2015). Restrictions of *ex vivo* organ models embrace ganglion cell death induced by axotomy, which leads to blockage of neurotrophin transport to the RGC bodies and thus shorter survival of the whole retinal draft (Kaempf et al., 2008; Osborne et al., 2001; Tezel, 2006).

Animal models, in which retinal hypoxia is induced, include photothrombosis of the retinal arteries with Rose Bengal (Kramer et al., 2009), increasing the intraocular pressure above systolic values (Selles-Navarro et al., 1996) and temporarily clamping the central retinal artery (Lafuente et al., 2002; Hayreh et al., 2004). However, animal experiments are restricted, very time-consuming, and expensive, and from an ethical point of view they should also be avoided. We therefore aimed to establish an *ex vivo* model to investigate retinal hypoxia using organotypic cultures. The model should be easy-to-use, cheap, reproducible and an alternative to animal testing.

## RESULTS

### Chamber-characteristics at 37°C

**Fig. 1.**
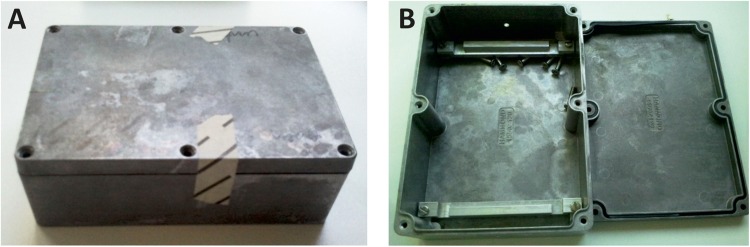
**The full-metal chambers with sealing ring are sealed with six screws.** (A) Chambers can be autoclaved and adjusted to the desired temperature in a reasonable time. (B) On both slim ends, small openings were drilled with exactly the size for standard 0.22 µm filters. The chamber is assembled under a sterile hood.

The here presented autoclavable full-metal chamber was the third generation of chambers we designed for this hypoxia model. The first two chamber types failed either to be air tight or adopt to temperature changes in a reasonable amount of time (Fig. 1).

**Fig. 2.**
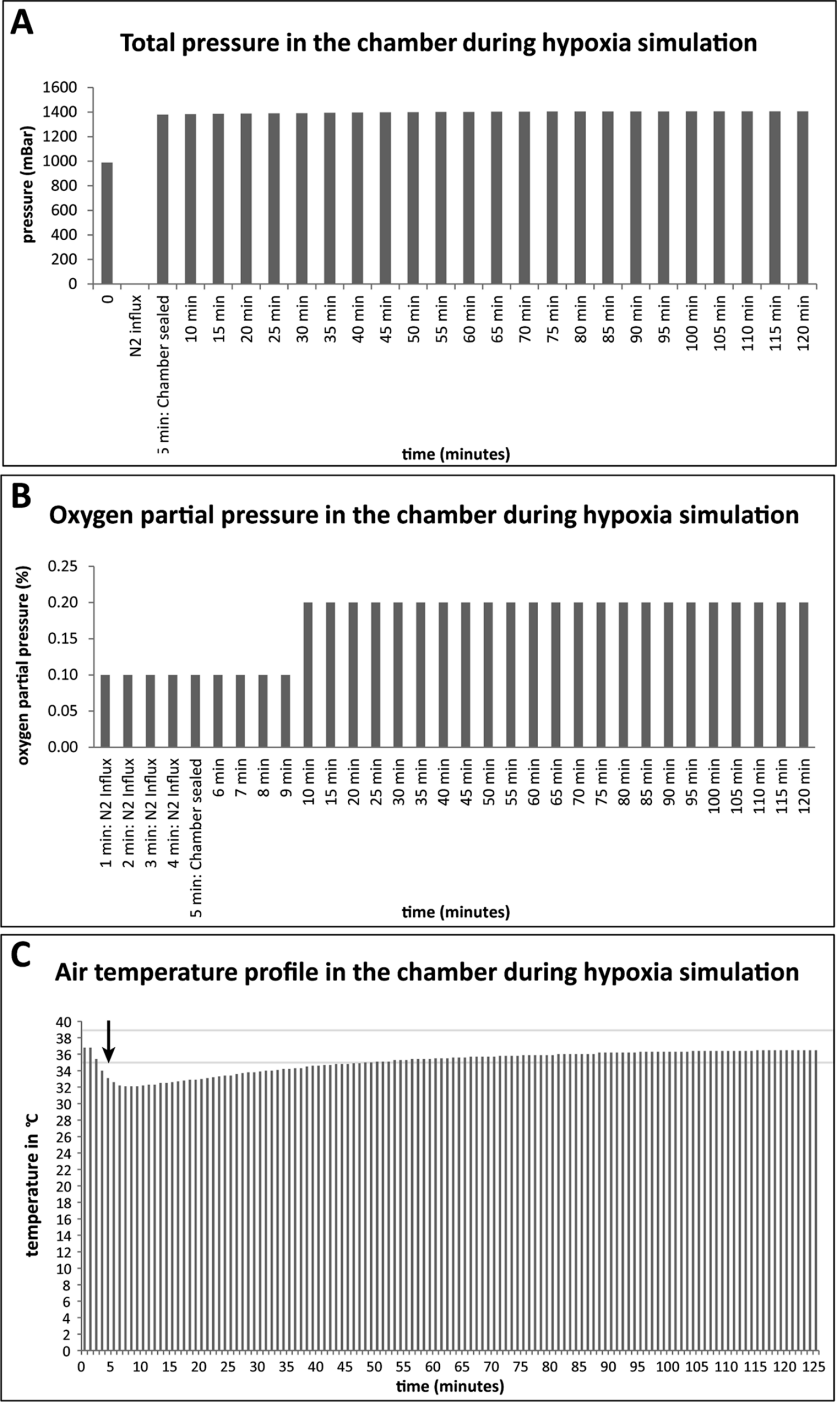
**Properties of the ischemia chamber.** The bar graphs show the measurements of the total pressure (A), the oxygen partial pressure (B) and the temperature inside the chamber (C). On the *x*-axis, the time is given in minutes (min). (A) Before any experiment, the chambers were adjusted with an open lid to the desired temperature for 12 h in a heating cabinet. Prior to sealing, the chambers were always inflated with N_2_ for 5 min. During the inflation, the total pressure could not be measured. Therefore, during that time bar graphs are missing. After sealing the chamber, the pressure is higher than the surrounding pressure and rises constantly, proving that the chamber is absolutely air tight. (B) The partial oxygen pressure increases slightly over 120 min due to solved oxygen inside the chamber. (C) Due to the gas influx for 5 min, the air in the chamber is cooled down for a short time to 32.2°C (arrow). After 48 min, the air inside the chamber reached the range of tolerance again (±2°C) (indicated by two gray lines).

Before the inflation is started, the pressures both inside and outside the chamber were equivalent (comparable to atmospheric pressure: 988 mBar) (Fig. 2A). To eliminate all oxygen from the chamber, it was streamed with N_2_ for 5 min. Then, the chamber was sealed at the opposite opening and immediately afterwards the influx of N_2_ was stopped, to prevent any influx from oxygen. After influx of N_2_ into the chamber, the pressure rose continuously from 1379 to 1407 mBar. Although the pressure is rather high, it is still on a level that is well tolerated since this pressure is equal to the pressure that acts upon a diver at a depth of 4 m. Furthermore, the pressure was only persisting during the hypoxia time (45-120 min); therefore, the sealed chamber was airtight. The partial oxygen pressure inside the full-metal chamber was reduced to 0.1% by N_2_-influx (Fig. 2B). 5 min after sealing the chamber the partial oxygen pressure rose to 0.2%. The partial oxygen pressure stayed stable at this level, until the end of observation (minute 120). However, one also needs to note that our measuring device showed only to one decimal place, therefore the rise might be only due to a very subtle change, which still caused the device to switch from 0.1 to 0.2. The temperature inside the chamber decreased continuously with the N_2_-influx from 36.8°C to 32.2°C. After sealing, the temperature inside the chamber rose steadily again from 32.2°C (minute 6) to 35.0°C (minute 48) and reached 36.5°C after 120 min of incubation. Nevertheless, 42 min after the end of the N_2_-influx the chamber reached the range of 37°C (±2°C) (Fig. 2C).

### Retinal ganglion cells and hypoxia

**Fig. 3.**
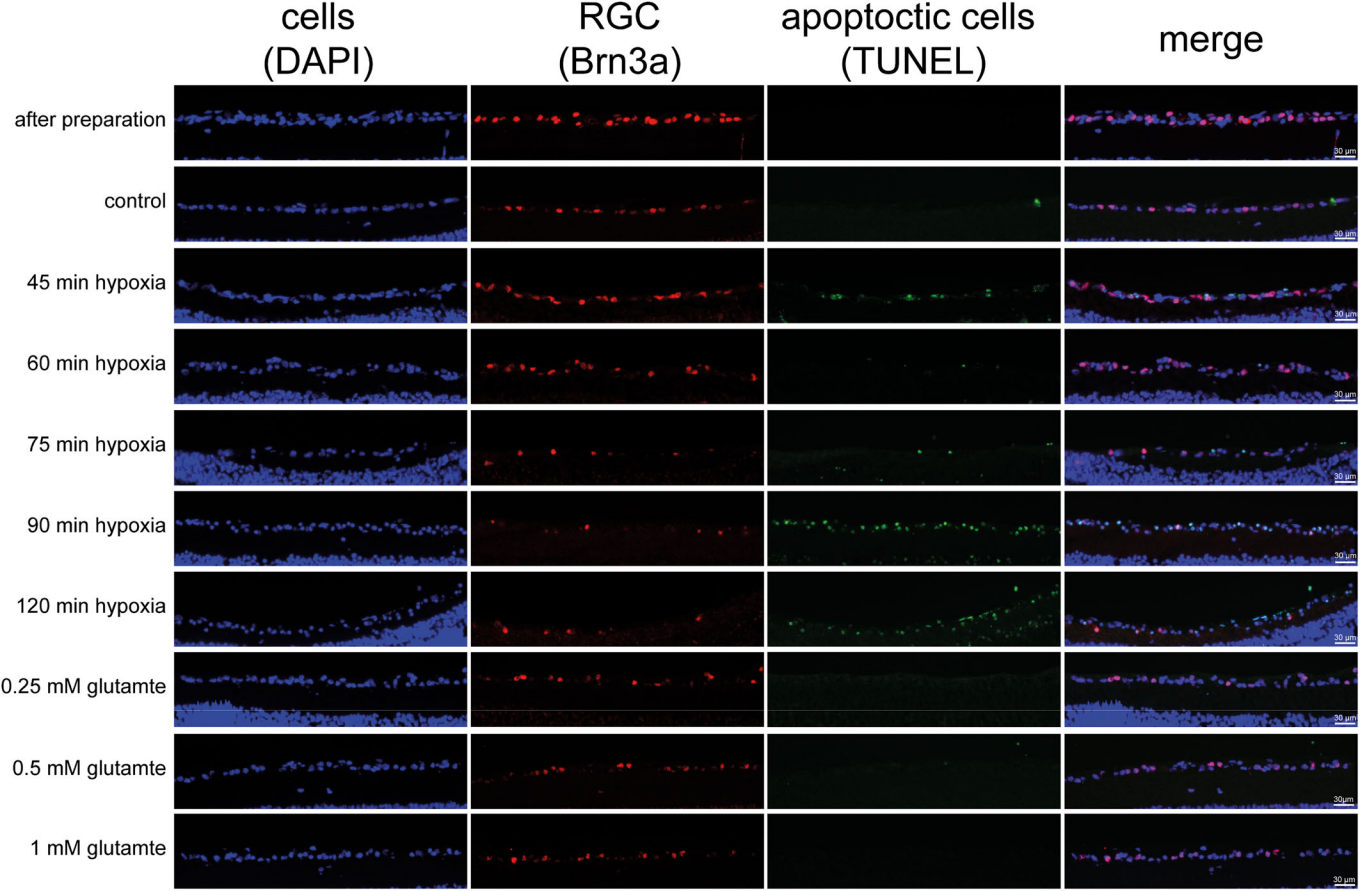
**Representative pictures of the retinal ganglion cell layer 48 h after different treatments with triple staining for cells (DAPI/blue), RGCs (Brn3a-antibody/red) and apoptotic cells (TUNEL-staining/green).** A decrease in the amount of RGCs and a rise in apoptotic cells in the ganglion cell layer is observable depending on ischemia duration. No TUNEL+ cells are found in immediately frozen retinas. However, additional cells in the retinal ganglion cell layer besides RGCs were found to be TUNEL+. Although glutamate causes a decrease in total cell amount and RGCs only very few TUNEL+ cells can be observed.

The number of RGCs visualized by Brn3a staining in the cultivated untreated retinas (control sample) is reduced compared to freshly prepared retinas [control: 24 h: 26.28±4.48%; 48 h: 25.65±11.15%; 72 h: 19.57±4.79%; retinas immediately after preparation: 38.43±4.16%; mean±standard deviation (s.d.)]. A clear time-dependent reduction of RGCs is obvious, resulting most likely from the axotomy of the RGCs. In the immediately frozen retinas no TUNEL-positive (TUNEL+) cells were found. Less RGCs were already detectable in the control groups, however no TUNEL+ cells were observed. In contrast, a time-dependent loss of RGCs and a rise in apoptotic cells in the ganglion cell layer was observable in retinas under hypoxic conditions. In addition, other cells than RGCs in the ganglion cell layer were stained positive for apoptosis. Although glutamate causes a decrease in total cell amount and RGCs, only very few TUNEL+ cells could be observed (Fig. 3).

**Fig. 4.**
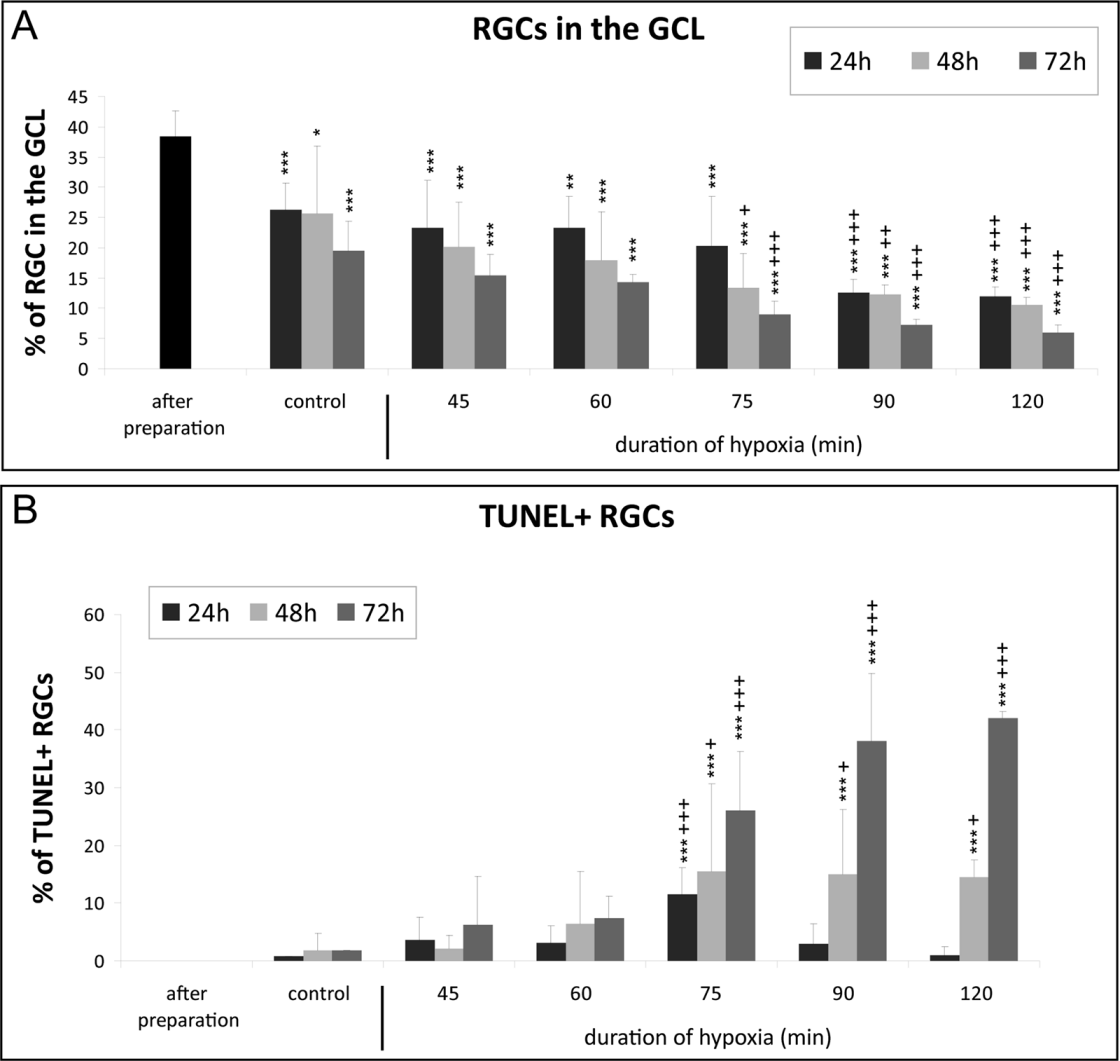
**Quantification of ischemia induced loss of retinal ganglion cells.** The bar graphs show the number of RGCs (A) and the number of TUNEL+ RGCs (B). (A) Quantification of the cells confirmed that the amount of RGCs compared to the total amount of cells in the retinal ganglion cell layer (GCL) is decreasing in a time- and hypoxia duration-dependent manner (indicated with *). From 75 min of hypoxia 48 h onwards, a significant difference is observable compared to the corresponding control (indicated with +). (B) Quantification of apoptotic cells showed that 75 min of hypoxia caused significant apoptosis compared to corresponding controls (indicated with +). The most significant results and ongoing rise in apoptotic cells were also achieved with 75 min of hypoxia (*n*=6 for hypoxia-treated retinas and *n*=10-20 for controls and ‘after preparation’). Data are depicted as mean±s.d., with *P<0.05, **P<0.01, ***P<0.001 versus ‘after preparation’; +*P*<0.05, ++*P*<0.01, +++*P*<0.001 versus control (ANOVA and Tukey's post hoc test); statistical differences between the groups were also revealed, however for the ease of reading are not given in the graph.

This decrease was hypoxia duration-dependent. For example, after 48 h of cultivation and 75 min of hypoxia the number of RGCs was significantly reduced compared to the control group after 48 h of cultivation (75 min N_2_ 48 h: 13.41±5.7% versus control 48 h: 25.65±11.15%; *P*<0.05). A shorter hypoxia duration did not result in a significant RGC loss compared to the corresponding control group after 24, 48 or 72 h of cultivation. Vice versa, a longer hypoxia duration resulted in a significant and stronger RGC loss than 75 min of hypoxia after 24, 48 and 72 h of cultivation (Fig. 4A).

In accordance with the reduced amount of RGCs, the number of TUNEL+ RGCs in general increased with a longer hypoxia duration. However, at the 24 h time-point the number of TUNEL+ RGCs was lower at 90 and 120 min than at 75 min. The amount of TUNEL+ RGCs was always significantly higher with 75 min of hypoxia compared to the corresponding control group. In detail, the amount of TUNEL+ RGCs after 24 h of cultivation was: 75 min N_2_:11.61±4.51% versus controls 0.82±1.00 (*P*=0.001), 48 h after cultivation: 75 min N_2_: 15.41±15.19% versus controls 1.88±2.89% (*P*<0.05) and 72 h of cultivation: 75 min N_2_: 25.98±10.28% versus controls 1.84±3.08% (*P*<0.001) (Fig. 4B).

### Retinal ganglion cells and excitotoxicity

**Fig. 5.**
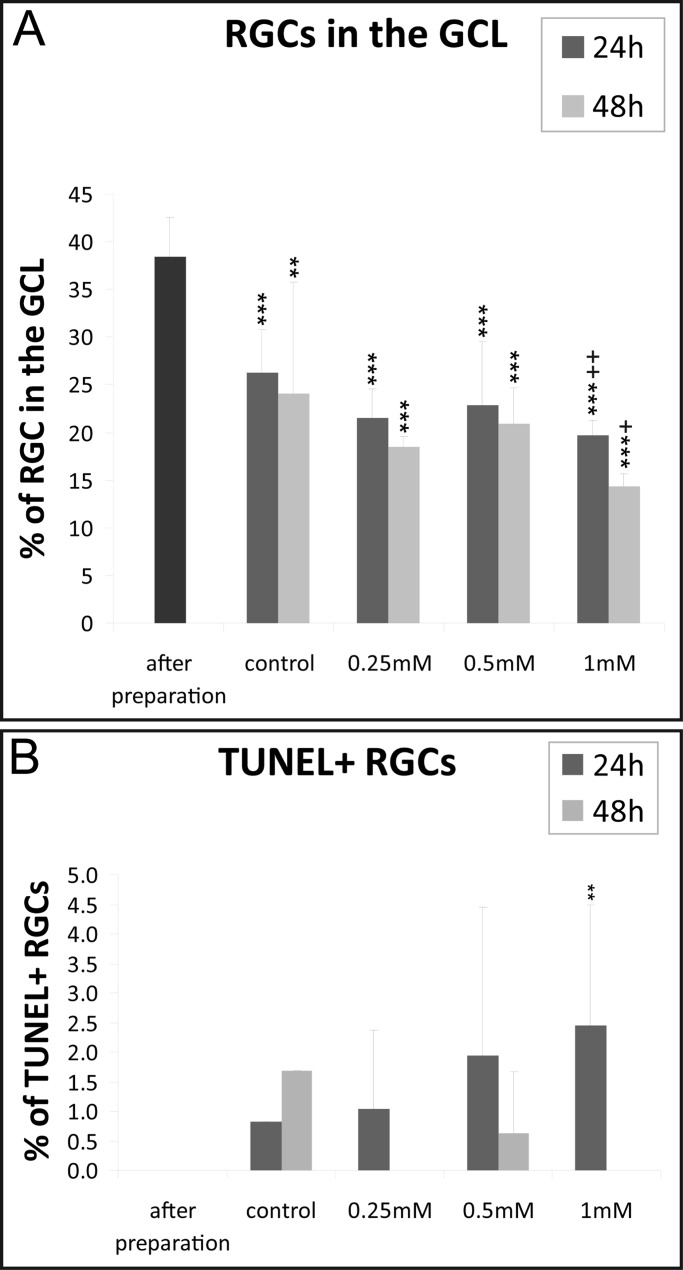
**Quantification of glutamate induced loss of retinal ganglion cells.** (A) Quantification of the cells confirmed that the amount of RGCs compared to the total amount of cells in the GCL is decreasing. 1 mM of glutamate was the first concentration that significantly differed from the corresponding controls. (B) In contrast, the amount of apoptotic cells was very low. Data are depicted as mean±s.d., with *P<0.05, **P<0.01, ***P<0.001 versus ‘after preparation’; +*P*<0.05, ++*P*<0.01, +++*P*<0.001 versus control (ANOVA and Tukey's post hoc test; *n*=6-7 for glutamate treated retinas and *n*=10-20 for controls and ‘after preparation’).

To validate the efficacy of the hypoxia model, the explants were also treated with different doses of glutamate (0.25, 0.5 and 1.0 mM). Compared to the controls only the retinas treated with 1 mM glutamate showed a significantly reduced number of RGCs after 24 h (controls: 26.28±4.48% versus 1 mM: 19.69±1.58%; *P*<0.01) and 48 h (controls: 24.04±11.68% versus 1 mM: 14.41±1.24%; *P*<0.05) of cultivation (Fig. 5A). Interestingly, the number of TUNEL+ cells only increased significantly after 24 h (2.45±2.04; *P*<0.01) but not anymore after 48 h (0.00±0.00) of cultivation with 1 mM glutamate (Fig. 5B).

### Retinal thickness

**Fig. 6.**
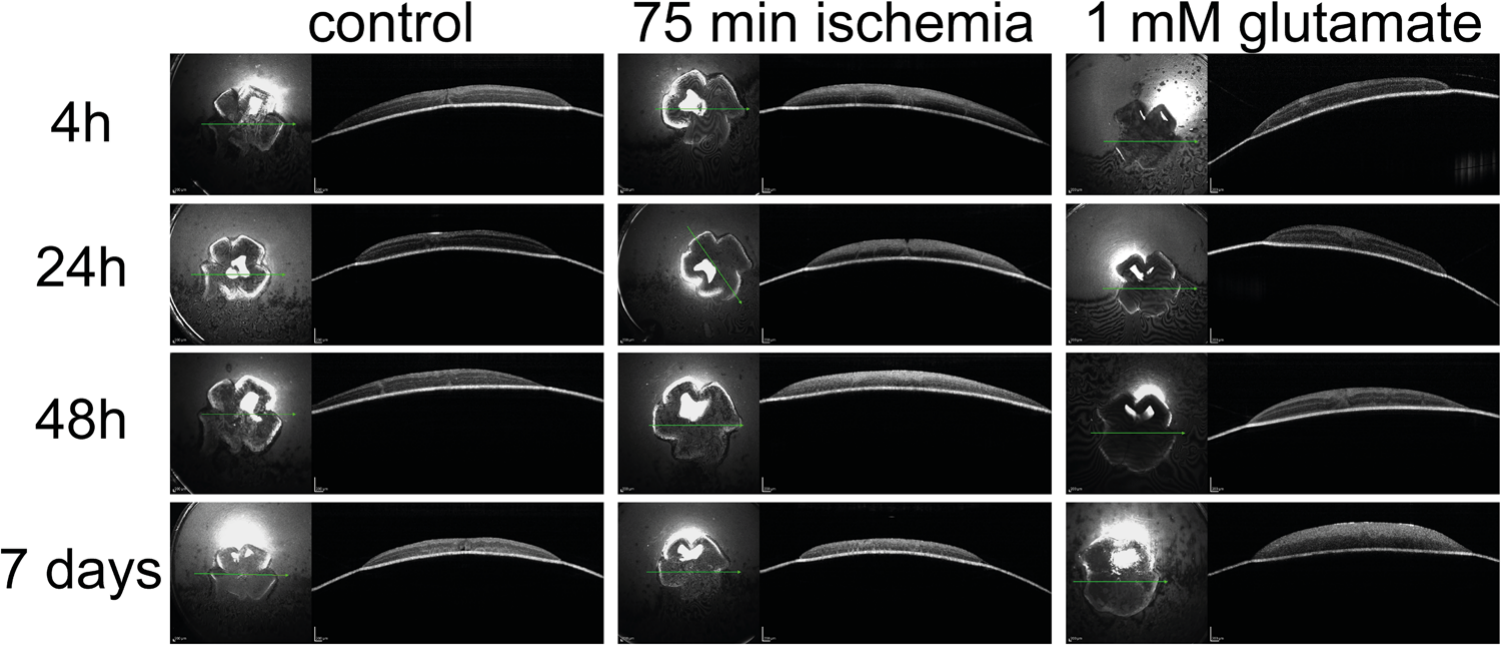
**Representative pictures of the OCT measurements: a time-dependent decrease in the retinal thickness was obvious in all cultures.** The layers of the inner retina could hardly be discriminated from each other, especially at later time-points, and showed hyper reflectivity with increased duration of hypoxia and incubation length.

**Fig. 7.**
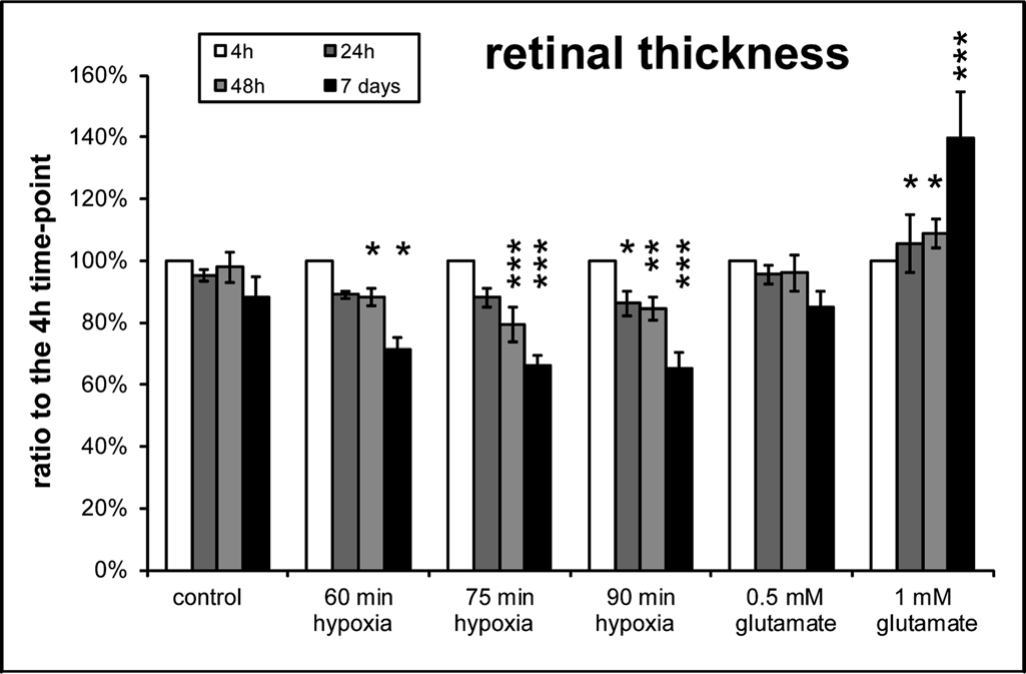
**Quantification of the retinal thickness proved a time- and hypoxia duration-dependent decrease.** In contrast high glutamate concentration was associated with a thickening of the retina (*n*=4 for all retinas, except 1 mM glutamate treated *n*=3). Data are presented with ±s.d. and *P<0.05, **P<0.01, ***P<0.001 versus control. Additional analysis with Tukey-Kramer post hoc analysis revealed more significant differences between the groups (for ease of reading this data is not shown).

Retinal thickness is a good indicator for the degenerative effects of hypoxia or excitotoxicity. Therefore, the retinal thickness was measured via optical coherence tomography (OCT) images. The clear advantage of this method is that the same retinas can be monitored over a desired time span. Only selected time-points from the prior experiments were further analyzed. Representative OCT pictures of the different treatments are given in Fig. 6. Quantification of the retinal thickness revealed that hypoxia resulted in a reduction and glutamate treatment in an increase of retinal thickness (Fig. 7). Compared to the corresponding control group the retinal thickness was significantly reduced from 60 min of ischemia and 48 h of cultivation onwards (all *P*<0.05) (Fig. 7). Only the groups treated with 90 min of ischemia showed a significantly reduced retinal thickness after 24 h of cultivation (*P*<0.05).

Interestingly, although the thickness of the retina was unchanged in all treatment groups 4 h after hypoxia, a distinction between the layers of the inner retina was more difficult with 75 and 90 min of hypoxia. This effect worsened with longer cultivation time. Furthermore, the inner retinal layers showed a hyper reflectivity when compared to the controls or to explants incubated under shorter hypoxia times. This was in contrast to the OCT images of the retinas treated with glutamate.

Congruently to the RGCs loss only the retinas treated with 1 mM glutamate resulted in a significant change of retinal thickness from 24 h of cultivation onwards (*P*<0.05) (Fig. 7). In contrast to the ischemia treated retinas, the distinction of the retinal layers was still easily possible at 24 and 48 h of cultivation. After seven days of cultivation, the retinal layers could be hardly distinguished from each other and the retinal thickness was further increased.

### Expression of β-tubulin

**Fig. 8.**
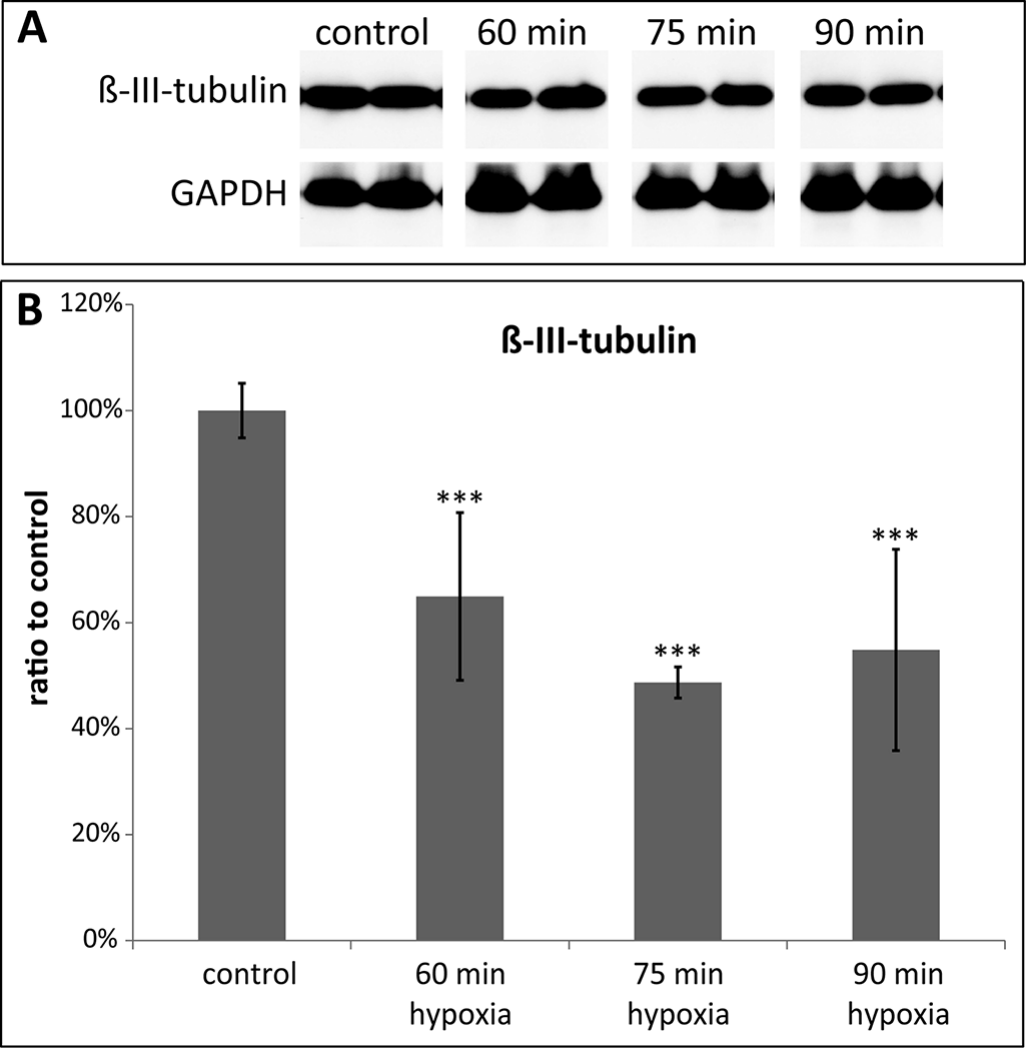
**Expression of TUBB3 protein.** (A) Representative western blot for protein expression analysis of β-III-tubulin. GAPDH served as loading control. (B) Quantification of western blot analysis confirmed that hypoxia in the ischemic chamber significantly reduced neuronal TUBB3 protein expression. Error bars indicate mean±s.d., *n*=4 for all treatment groups. Statistical significance is indicated as * with respect to the control condition using the following significance level: ***P<0.001 (ANOVA und Dunnett's post hoc test).

β-III-tubulin is a distinct marker for RGCs (neurons). To finalize the exact model properties (hypoxia duration), we decided to further focus on the 48 h of cultivation as from the prior experiments this seemed to be the best cultivation time. Western blot analyses were therefore only performed after 48 h of cultivation and with only three selected time-points from prior experiments (Fig. 8A). The β-III-tubulin expression decreased significantly after 60, 75 and 90 min of hypoxia compared to the control group after 48 h of cultivation (controls: 100±18.95%; 60 min N_2_: 64.94±5.16%; 75 min N_2_: 48.69±15.81%; 90 min N_2_: 54.84±2.94%, all *P*<0.001) (Fig. 8B).

## DISCUSSION

### Model development

We successfully established a reliable, reproducible and easy-to-use organotypic culture model for retinal hypoxia with our self-designed ischemia chamber. The sealed chamber is airtight, as the overall pressure inside the chamber increases continuously after sealing of the chamber. The increase of the partial oxygen pressure from 0.1% to 0.2% is most likely caused by resolved oxygen from the medium. No color change of the media was observed after applying nitrogen, therefore, it can be assumed that the pH value of the medium remained stable or did not change more than 1.5 units.

Standard culture medium (not-deoxygenated) was used to establish this model. As a result, a severe hypoxia was only induced in the inner retina, as the culture medium could still supply a limited amount of oxygen to the photoreceptors and the outer part of the retina throughout the ischemic treatment. Our experimental set up therefore resembled the clinical situation, where only the inner retina suffers from hypoxia as in ischemic diabetic retinopathy, central retinal artery occlusion and ischemic retinal vein occlusion.

### RGC death and comparison to *in vivo* models

Dependent on the duration of hypoxia the amount of RGCs decreased after 24, 48 or 72 h. The damage to RGCs produced by 75 min of hypoxia was comparable to the damage of 1 mM glutamate incubation (G) after 24 h (75 min N_2_: 20.27% versus 1 mM G 19.69%) and 48 h (75 min N_2_: 13.41% versus 1 mM G: 14.41%) of cultivation. The loss of RGCs in the control group is, most likely, due to the axotomy of the optic nerve which is an unavoidable fact in organotypic retinal cultures (Nadal-Nicolás et al., 2015b).

In contrast, the amount of TUNEL+ RGCs was also dependent on hypoxia duration, however in glutamate-treated retinas only few apoptotic RGCs were found and the difference between the treated groups and the controls only reached significance in the 1 mM group after 24 h. An explanation of this fact might be that either the RGCs die via a non-apoptotic pathway, or the chosen time-points are too long. Xin et al. reported that the number of TUNEL+ cells in the ganglion cell layer increased in a dose-dependent manner in postnatal mouse retina 18 h after glutamate treatment (Xin et al., 2007). However, these retinae were cultured seven days before treatment and the glutamate concentration used was lower (10 µM-100 µM). Therefore, it might be that the glutamate-induced damage in our retinae is faster and the apoptotic cells have already been cleared.

In addition, in the OCT measurements hypoxia led to a significant decrease in retinal thickness and 1 mM glutamate caused a thickening of the retina. Therefore, the loss of RGCs was comparable between the two methods, but the effect on the tissue was different.

As ocular nerve transection does not lead to RGC cell death during the first three days (Lafuente et al., 2002; Berkelaar et al., 1994; Peinado-Ramon et al., 1996), the loss of RGCs in our untreated controls should be mainly due to the culture conditions or preparation damage.

Although in *in vivo* experiments reperfusion leads to an additional tissue damage (Bulkley, 1987), toxic and pro-apoptotic factors like glutamate are also washed out. Considering this, it is not surprising, that in our model the amount of dying RGCs is a little bit higher than in the reported animal models. For instance, Selles-Navarro et al. reported that even five days after a pressure-induced CRAO for 75 min, 67% of the RGCs were still alive (Selles-Navarro et al., 1996). Another publication from the same lab, however, with another method of CRAO (induction by ligature of the ophthalmic vessels), even showed a RGC survival of 75% five days after a CRAO of 90 min (Lafuente et al., 2002). In contrast, Zhu et al. observed a 53% loss of cells in the ganglion cell layer with pressure elevation for 45 min after one week (Zhu et al., 2002). These differences could be explained by the different techniques and retrograde tracers the groups employed to identify surviving RGCs (Nadal-Nicolás et al., 2015a).

### Changes in retinal thickness

The fact that retinal hypoxia resulted in retinal thinning after only a few days was rather surprising. In the clinical setting, the inner retina is thicker for several weeks after acute ischemia caused by central retinal artery occlusion. One month after central retinal artery occlusion the atrophy of the retina leads to ‘pseudo-normalization’ and finally ends in a clearly atrophic retina three months after the insult (Cornut et al., 2012). The difference between our model and central retinal artery occlusion in humans is that in our model hypoxia ends already after 120 min, whereas the central retinal artery occlusion including the ischemic situation continues. In addition, retinas with a central retinal artery occlusion for 120 min exhibit nearly no swelling in the OCT and normalize very quickly after reperfusion. Therefore, the cells surviving the ischemia duration do not continue swelling as in an ongoing ischemic insult. As a consequence, only very sensible cells like RGCs die and the retina becomes thinner. Another explanation for the missing initial swelling of the retina might be that, in our *ex vivo* model, no perfusion of the retina exists and therefore no vasogenic edema or angiogenesis develops (Shortt et al., 2004; Zhu et al., 2002). Under ischemic conditions *in vivo*, fluid leaks out from damaged capillaries due to a dysfunction of the blood-brain-barrier, causing retinal swelling (Kaur et al., 2007). Probably these two mechanisms are responsible for the retinal thinning in our model after the hypoxia treatment. Interestingly, as in the clinical situation, the layers of the hypoxic inner retina could hardly be discriminated from each other and the inner plexiform and inner nuclear layer showed hyperreflectivity with increased duration of hypoxia. These are typical signs observed in OCT scans of retinal ischemia or hypoxia in humans (Chu et al., 2013; Coady et al., 2015).

In contrast to the hypoxic insult the glutamate treatment was performed for 24 h, rather than only for up to 120 min. After 24 and 48 h, the layers could be easily distinguished even with 1 mM glutamate treatment, but not after seven days of cultivation. This fact, as well as the increase of retinal thickness, is a clear sign for retinal damage. The same effect has been recently descripted by Rovere et al. (2015) after intraorbital optic nerve transection (IONT). In adult rats a transient swelling of the retinal thickness was observed until day twelve. This observation was explained by a transient general inflammatory response following ON injury, including macro- and microglial activation (Rovere et al., 2015).

### Hypoxia duration selection for further studies

For further studies, we selected 75 min of hypoxia and a cultivation time of 48 h. These conditions cause approximately 50% RGC death compared to the untreated control explants after 48 h. At this time-point, an additional 15% of RGCs were also apoptotic and the retinal thickness was reduced significantly. A death rate of 50%, like in our model, should be a suitable and sensitive experimental set-up for evaluation of neuroprotective agents.

### Conclusions

An easy-to-use *ex vivo* retinal hypoxia model was introduced that reliably induced retinal damage on a morphological (retinal thickness), and molecular (protein expression and apoptotic markers) level. The best time-point to investigate effects was identified as 48 h after preparation of the culture. The maximum limit of this culture method using stress agents is seven days of cultivation. 75 min of hypoxia was selected as the best hypoxia duration for further studies, as approximately 50% of the RGC died and 15% of RGCs were apoptotic. *Ex vivo* studies with neuroprotective agents will be performed to further affirm the quality of this model.

With this model, several therapies or neuroprotective agents against ischemic injury can be tested *ex vivo* prior to animal experiments. Therefore, the amount of animal experiments can be reduced. Furthermore, the results of this organotypic organ culture model are superior to the results received with primary cell cultures.

## MATERIALS AND METHODS

### Materials

R16-basal-medium and R16-complete medium were prepared as described previously (Schultheiss et al., 2016).

### Chamber development

First, we developed a hypoxia chamber. However, the first two versions of the chambers did not fulfill the demanded standards. Therefore, we developed an autoclavable full-metal chamber with small holes on the shorter sides for the connections of the gas tubes (Fig. 1). We tested several parameters of the chamber in order to be sure that the chamber was airtight and could adapt fast to temperature changes. The partial oxygen pressure (Vacuum Barometer and Manometer GDH 200-14; Greisinger electronic GmbH, Germany), the overall pressure (Oxygen Meter GOX 100; Greisinger electronic GmbH, Germany) and the temperature (Temperature Logger EBI 20, Ebro, Germany) were measured. Before any experiment, the chambers were adjusted with an open lid to the desired temperature for 12 h in a heating cabinet.

### Culture preparation

Male and female Lister-Hooded rats (*Rattus norvegicus*), 14-15 days postnatal (Charles River, Germany), were euthanized with carbon dioxide inhalation. Animals were treated and euthanized according to German animal protection guidelines. Eyes were enucleated immediately after death, washed in ethanol, PBS and R16-basal medium. Then the eyes were transferred under a sterile hood and the retinas were prepared in R16-basal medium. After preparation, the retinas were transferred on culture plate inserts (Transwell Permeable Supports 0.4 μm polyester Membrane, 6-well plate, Coster, USA) with the ganglion cell layer up. R16-complete medium was added to the wells. The medium was exchanged the first day in culture and every second day thereafter. Preparation time was on average 12 min (range: 10-15 min) from death until both retinae were with media in the 6-well plate (Fig. 3). To assure a similar preparation standard and exclude hypoxia during preparation, an overall time limit and a time limit for each step was applied. Retinas exceeding these time limits were not included in this study but used for antibody evaluation. Control explants were transferred immediately after preparation to an incubator with standard conditions without further treatment (*n*=10-20). Additional cultures were immediately frozen without treatment to evaluate the RGC loss in the controls.

### Hypoxia treatment – incubation with nitrogen

The ischemia chambers with the organotypic cultures in a 6-well plate were streamed with N_2_ for five minutes, then the chambers were immediately sealed and the retinas were incubated for the rest of the designated time (45–120 min). After the incubation, the 6-well plate was removed from the chamber and left under a sterile bench with no lid for two minutes to adjust the air in the well plate to normal conditions. Next, the 6-well plates were incubated in an incubator at 37°C in an environment containing 5% CO_2_. Sample size was *n*=6.

### Glutamate treatment

To the glutamate-treated cultures, R16-complete media with the desired concentrations of glutamate (0.25-1 mM glutamate) instead of R16-complete medium was added. After 24 h of incubation, the media was exchanged to R16-complete media without glutamate. The glutamate-treated retinas were incubated at 37°C in an environment containing 5% CO_2_. Sample size was *n*=6-7.

### Immunohistochemistry

Frozen retinas were cut on a cryostat (12 µm sections). Triple staining with 4′,6-Diamidin-2-phenylindol (DAPI; 4′6′-diamidino-2-phenylindole; Invitrogen, Germany), Brn3a-antibody (SC-31984, Santa Cruz, USA) and TdT-mediated dUTP-biotin nick end labeling (TUNEL) were performed to determine the total cell amount in the ganglion cell layer, RGCs and apoptotic cells. First, cells were fixed with 4% paraformaldehyde. TUNEL staining was performed with the *In-Situ* Cell Death Detection Kit, Fluorescein, (Roche, Germany) according to manufacturer's protocol. Immediately afterwards sections were blocked in 5% PBS and the Brn3-antibody (1:866; Santa Cruz, USA) was applied overnight at 4°C. Then sections were washed, the secondary antibody (Cy3 Rabbit anti-goat; 1:2000; #305-167-003 Jackson ImmunoResearch, Germany) was applied for 90 min at room temperature, washed, counterstained with DAPI, washed again and mounted. Pictures were taken using a fluorescent microscope (Axioplan 2 Imaging; Zeiss, Germany) and cells were counted manually by a masked investigator.

### OCT and infrared measurements

The OCT measurements were performed as described previously (Schnichels et al., 2016). Cultures were briefly investigated immediately (3-4 h after preparation), 24 h, 48 h and 7 days after preparation. Flat-mounts were placed in a custom-made mounting device in a 90° angle in front of a Spectral-Domain-OCT. According to the infrared-overview 30°-line scans (ART: 100) were performed. Additional volume scans were performed for better orientation. The retina cultures were kept under aseptic conditions through the whole procedure. For quantification of the retinal thickness, the middle of sections was determined and measured. Additionally, from the middle three equidistant measurements were performed per side, resulting in seven measurements per picture. For each retina, three different pictures were quantified. The pictures were selected by a masked investigator and measured by a second masked investigator.

### Western blot

48 h after explantation, retinas were collected, homogenized and protein was isolated from the cells using cell extraction buffer (Invitrogen, Germany) containing Protease Inhibitor Cocktail Set III (Calbiochem, Germany) and 1 mM phenylmethylsulfonylfluorid (PMSF). Protein concentration was determined by the BCA Protein Assay Kit (Pierce, Germany). Equal amounts of protein (10 µg) were loaded onto 12% SDS gels, followed by wet-transfer with Towbin-Buffer to nitrocellulose membranes. SDS-PAGE and wet-transfer was performed using standard protocols (Schnichels et al., 2011). Immunostaining was carried out with a β-III-tubulin antibody (1:1000; MAB1195, R&D Systems, Germany); a GAPDH antibody (1:5000) was used for normalization. Secondary antibodies were peroxidase-conjugated. All antibodies (except for β-tubulin) were purchased from Cell Signaling, Germany (GAPDH: 2118, 2nd antibodies: #7076, #7074). Chemiluminescence was detected using the ECL chemiluminescence system (ThermoFisher Scientific, Germany). Protein ratios were calculated based on densitometrical quantification of scanned films using ImageJ (NIH) with four independent experiments (*n*=4). Protein levels were measured three times per experiment. Protein levels were normalized to GAPDH levels and one control. Controls were then set as 1 considering error propagation of the standard deviations.

### Statistics

Statistical analysis and sample size calculation was performed using JMP^®^ (SAS Institute Inc., Cary, NC, USA). ANOVA and Tukey's post hoc test were used to test for differences between the groups in the antibody staining and the western blot analysis. ANOVA und Dunnett's post hoc test were used to test for differences between the groups in the OCT measurements. Differences were considered to be significant at *P*<0.05. Retinas were excluded if visible damage to them occurred during preparation or any other procedure before fixation. In addition, all sections were first assessed via DAPI staining. If the DAPI staining showed severe damage to the section, the section was excluded from data evaluation. Retinas were masked after the final day of cultivation by S.S. In addition, the examiner at the OCT was also masked (T.D., F.Z.). All experiments and analysis (histology, western blot and OCT) were conducted by masked investigators (M.B., J.W., J.H.). After unmasking, the data was analyzed by M.B. and S.S.
